# Bidirectional talk between AhR and Oct4

**DOI:** 10.18632/oncotarget.4633

**Published:** 2015-06-24

**Authors:** Bo Kang, Ying-Jie Wang

**Affiliations:** State Key Laboratory for Diagnosis and Treatment of Infectious Diseases, Collaborative Innovation Center for Diagnosis and Treatment of Infectious Diseases, the First Affiliated Hospital, School of Medicine, Zhejiang University, Hangzhou, Zhejiang, China

**Keywords:** Chromosome Section, embryonic development, transcriptional regulation, AhR, Oct4

The aryl hydrocarbon receptor (AhR) is a member of the family of basic helix-loop-helix transcription factors that is widely expressed in most differentiated mammalian cells and implicated in a variety of cellular processes, such as cell cycle progression, cell migration, carcinogenesis and immune functions. Upon the binding of its synthetic or natural ligands, the cytosolically-located AhR is activated and translocated into the nucleus where it dimerizes with ARNT (the AhR nuclear translocator, or HIF-1β) and specifically binds to a consensus DNA sequence (5′-TNGCGTG-3′) known as an AhR-responsive element (AHRE) or dioxin response element (DRE), leading to changes in target gene transcription. The best characterized high-affinity ligands for the AhR are synthetic halogenated aromatic hydrocarbons and polycyclic aromatic hydrocarbons such as 2,3,7,8-tetrachlorodibenzo-p-dioxin (TCDD), and a growing number of natural products such as arachidonic acid metabolites, indoles, tetrapyrroles and polyphenolics are found to be able to act as AhR ligands as well. However, the physiological or pathological relevance of those natural ligands remained to be fully addressed [1].

Considerable AhR target genes identified by genome-wide gene expression profiling have morphogenetic functions, indicating that AhR may play a role in embryonic development. In fact, the developmental toxicity of TCDD has been well documented. By RNA sequencing-based analysis, it was implicated that the overall effect of TCDD-driven AhR activation was to maintain the pro-proliferative state of mouse embryonic stem cells (ESCs) and inhibit their differentiation into cardiomyocytes. Remarkably, in TCDD-treated AhR-positive differentiating ESCs, key pluripotency-associated transcription factors such as Oct4, Sox2, Nanog, Klf4 were all predicted to be activated at the transcriptional level [2], indicating a potential connection between AhR signaling and the core pluripotency factors.

The results from a subsequent study showed that AhR expression was maintained in a repressed but poised state in mouse ESCs which allowed for its quick activation during differentiation. Such repression resulted from the direct binding of the Oct4-Nanog-Sox2 complexes onto the AhR distal promoter region, along with PcG-mediated repression and pausing of unproductive RNA polymerase II molecules on the TSS (transcription start site) proximal promoter region. Release of the core pluripotency factors (particularly Oct4) from their binding sites can switch AhR expression from repression to activation [3]. This indicated that one of the previously unappreciated roles of Oct4 in ESCs is to transcriptionally repress AhR to maintain pluripotency. In this context, it is of note that repressed AhR activities may also be important for the multipotency of adult stem cells, since its synthetic antagonist StemRegenin 1 can promote the self-renewal and expansion of hematopoietic stem cells [4] and leukemic stem cells [5].

Recently, we showed that, compared to differentiated human cell lines, human ESCs and embryonal carcinoma cells (ECCs) possess the highest Oct4 mRNA levels yet the lowest AhR mRNA levels, and among various normal human tissues, placenta derived from Oct4-deficient trophectoderm exhibited the highest level of AhR mRNA. During RA-induced differentiation of human ESCs and ECCs, the Oct4 mRNA level decreased concurrently with an increase in AhR mRNA level, implicating a strong negative correlation between these two factors at the transcriptional level [6]. Such observation may be explained by the relief of suppressed AhR expression with reduced Oct4 as concluded from the above-mentioned study [3]. However, our further work revealed a reverse connection between AhR and Oct4 that may offer an alternative interpretation. By taking a combinatorial approach, we discovered that AhR can specifically bind to the AHRE motif (−499 ~ −495 relative to TSS) in the Oct4 (encoded by *POU5F1* gene) promoter *in vivo* and act as a transcriptional repressor of *POU5F1* [6]. Such repression was mediated by an endogenous AhR ligand, 2-(1′H-indole-3′-carbonyl)-thiazole-4-carboxylic acid methyl ester (ITE), one of the derivatives of tryptophan [7] that possessed anti-tumor efficacy *in vitro* and *in vivo* [6]. In various experimental settings, ITE was found to inhibit Oct4 transcription via strengthening the suppressive binding of AhR to the *POU5F1* promoter, and such effect appeared to be specific for ITE but not other tested tryptophan derivatives [6].

Thus, these new findings raise an interesting possibility of reciprocal suppression between AhR and Oct4. The chicken-and-the-egg question then follows: who plays a leading role in differentiation? Since Oct4 is highly expressed while AhR is undetectable in ESCs, it is likely that a reduction in the Oct4 level may come ahead of an increase in the AhR level. Once a small number of AhR molecules are generated, they can bind to the Oct4 promoter and inhibit its transcription in a ligand-specific manner, eliciting a feed-forward effect that drives accelerated reduction of Oct4 and differentiation. In such a postulated model, the concentration of the Oct4-suppressive ligands (such as ITE) may be a key accessory factor that regulates the progression of differentiation. In future studies, it will be important to fully decipher the cross-talk between AhR and Oct4, and how it is regulated by tryptophan-derived ligands and other endogenous AhR ligands in the context of embryonic development and lineage-specific differentiation.

## References

[1] Murray IA (2014). Nat Rev Cancer.

[2] Wang Q (2013). Environ Health Perspect.

[3] Ko CI (2014). Stem Cell Res.

[4] Boitano AE (2010). Science.

[5] Pabst C (2014). Nat Methods.

[6] Cheng J (2015). Nat Commun.

[7] Song J (2002). Proc Natl Acad Sci U S A.

